# Construction of a Risk Assessment Model for Short-Term Mortality in Patients with Invasive Fungal Diseases Post-Cardiac Surgery Based on Multivariate Analysis

**DOI:** 10.3390/pathogens14111116

**Published:** 2025-11-03

**Authors:** Dong Wei, Qi Shen, Qian Zhai

**Affiliations:** Department of Cardiac Surgery Intensive Care Unit, Qilu Hospital of Shandong University, 107 Wenhua Xilu, Jinan 250012, China; patrick1229@mail.sdu.edu.cn (D.W.); shenqi@email.sdu.edu.cn (Q.S.)

**Keywords:** invasive fungal diseases, cardiac surgical procedures, nomogram, risk assessment, LASSO-logistic regression, Mycoses, intensive care units, mortality

## Abstract

To develop and validate a predictive model for assessing the risk of short-term mortality in patients with invasive fungal diseases (IFDs) following cardiac surgery. This retrospective study analyzed clinical data from patients diagnosed with postoperative IFDs in the cardiac surgical intensive care unit (ICU) of Qilu Hospital of Shandong University (QLH), between January 2020 and December 2023. A total of 98 patients were included and divided into a non-survival group (*n* = 42) and a survival group (*n* = 56) based on 28-day mortality. Demographic, clinical, and postoperative parameters were collected. The Least Absolute Shrinkage and Selection Operator (LASSO) regression was used for variable selection, and selected variables were then entered into multivariate logistic regression to identify independent risk factors. A nomogram was developed, and its predictive performance was evaluated using the receiver operating characteristic (ROC) curve, decision curve analysis (DCA), and clinical impact curve (CIC). Multivariate logistic regression, following variable selection by LASSO, identified a history of smoking, an elevated SOFA score, mean arterial pressure (MAP) below 70 mmHg, and tachyarrhythmia as independent risk factors for short-term mortality in this cohort (*p* < 0.05). The prediction model demonstrated excellent discrimination, with an area under the ROC curve (AUC) of 0.886 (95% CI: 0.816–0.957). The calibration curve showed good agreement between predicted and observed outcomes, with a mean absolute error of 0.023. Decision curve analysis indicated a net clinical benefit across a threshold probability range of 0.1 to 0.87. The clinical impact curve confirmed a high concordance between predicted mortality and actual outcomes. A history of smoking, an elevated SOFA score, MAP below 70 mmHg, and tachyarrhythmia independently predict short-term mortality in patients with IFDs after cardiac surgery. Therefore, the nomogram constructed from these factors provides an accurate and clinically applicable tool for risk stratification.

## 1. Introduction

Infection has long been recognized as a significant complication following surgical procedures, constituting one of the primary adverse factors hindering postoperative recovery [1]. In cardiac surgery, the reported overall postoperative infection rates range from 3.5% to 26.8% [2], significantly higher than those observed in general surgical procedures. Although the overall incidence of IFDs after cardiac surgery is relatively low, approximately 1–3% [3], it is associated with extremely high short-term mortality. Multicenter studies have demonstrated that the short-term mortality rate in cardiac surgery patients with postoperative candidemia can reach up to 53% [4]. The distinctive pathophysiological features of IFDs often render early clinical symptoms nonspecific, and substantial surgical trauma combined with the immunosuppressive effects of cardiopulmonary bypass frequently leads to delayed diagnosis. Consequently, IFDs have emerged as a critical contributor to postoperative mortality in cardiac surgery patients.

Although diagnostic criteria for IFDs have been progressively refined by the EORTC/MSGERC 2020, the EORTC/MSGERC 2021 ICU Working Group, and the FUNDICU 2024 consensus statements [5,6,7], these guidelines have primarily focused on hematologic disorders and general critical care populations. A systematic understanding of the risk factors and short-term prognosis specifically for cardiac surgery patients remains inadequate, and currently, no validated predictive tool for 28-day mortality is available for this patient subgroup. To address this gap, we retrospectively analyzed cases of postoperative IFDs following cardiac surgery in a single-center setting. Independent risk factors were identified using LASSO regression, and a predictive nomogram model was subsequently constructed based on multivariate logistic regression. This model aims to quantify the risk of short-term mortality, thereby providing evidence-based guidance for perioperative management and improving patient outcomes.

## 2. Materials and Methods

### 2.1. Study Population

**Figure 1:**
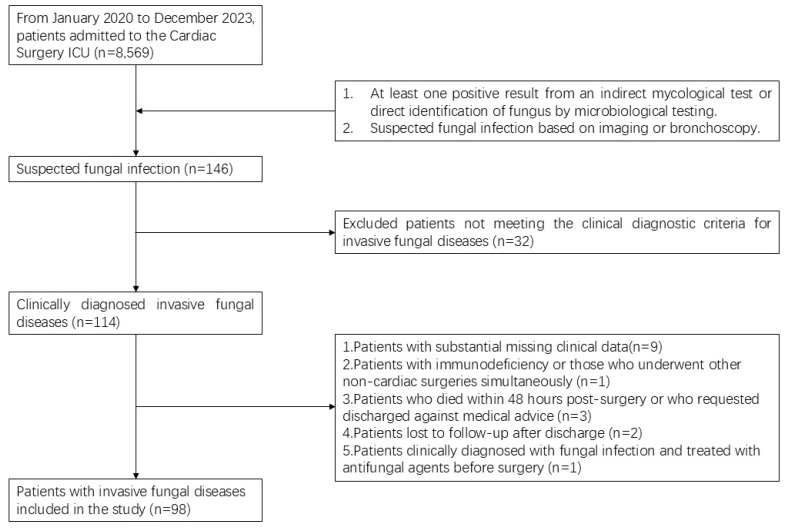
Flow chart of patient inclusion.

This study retrospectively analyzed patients with suspected fungal infections admitted to the cardiac surgery ICU of QLH, from January 2020 to December 2023. A total of 146 postoperative cases were initially identified. After excluding patients with incomplete medical records, patients lost to follow-up, or confirmed preoperative fungal infections, 98 adult patients who met the clinical diagnostic criteria for IFDs remained. These patients were included in the final analysis after a consensus review by two experienced clinicians. Based on their 28-day postoperative outcomes, patients were divided into a survival group (*n* = 56) and a non-survival group (*n* = 42) (Figure 1).

### 2.2. Diagnostic Criteria

#### 2.2.1. Probable Invasive Fungal Disease

All cases were initially evaluated according to the EORTC/MSGERC 2020 criteria [7]. They were then re-assessed using the FUNDICU 2024 criteria [6]. For critically ill ICU patients, a diagnosis of probable invasive pulmonary aspergillosis (IPA) or tracheobronchial aspergillosis (TBA) required at least one host factor and one clinical feature. Clinical features included worsening respiratory insufficiency despite appropriate antibiotic therapy and ventilatory support; persistent fever (≥38.3 °C) lasting ≥3 days despite adequate antibiotic therapy; or hemoptysis. This diagnosis also required at least one mycological criterion. These included bronchoscopic findings such as ulcers, nodules, pseudomembranes, plaques, or eschars, or a positive microbiological test result.

#### 2.2.2. Confirmed Candidemia

Confirmed candidemia was defined as the isolation of *Candida* spp. from at least one blood culture obtained via venipuncture (non-catheter source).

### 2.3. Inclusion and Exclusion Criteria

#### 2.3.1. Inclusion Criteria

Patients who have undergone cardiac surgery and meet the aforementioned diagnostic criteria for invasive fungal diseases, as independently confirmed by two senior clinicians.

Patients aged 18 years or older.

#### 2.3.2. Exclusion Criteria

Patients with substantial missing clinical data.

Patients with preoperative immunodeficiency or those undergoing concurrent non-cardiac surgery.

Patients who died within 48 h post-surgery or who requested discharged against medical advice prior to clinical recovery.

Patients lost to follow-up after hospital discharge.

Patients clinically diagnosed with fungal infection and treated with antifungal agents before surgery.

### 2.4. Clinical Data Collection

Clinical data were extracted from electronic medical records, including: (1) Baseline characteristics: age, sex, body mass index (BMI), history of smoking and alcohol consumption, comorbidities, New York Heart Association (NYHA) functional class, left ventricular ejection fraction (LVEF), and pulmonary artery systolic pressure. (2) Perioperative details: preoperative diagnosis, type of surgery, use and duration of cardiopulmonary bypass (CPB), intraoperative blood loss, and transfusion of blood products. (3) Severity scores at IFDs diagnosis: Sequential Organ Failure Assessment (SOFA) score and Acute Physiology and Chronic Health Evaluation II (Apache II) score. (4) Laboratory and clinical parameters: The earliest positive pathogenic specimen for IFDs was identified. Serum galactomannan (GM) was determined using an enzyme immunoassay kit (Platelia™ Aspergillus Ag ELISA, Bio-Rad Laboratories, Marnes-la-Coquette, France). Serum (1,3)-β-D-glucan (G test) was measured using a diagnostic kit (Danna Biological Technology Co., Ltd., Zhuhai, China). Both GM and G tests were performed with a microplate reader (Thermo Scientific, Waltham, MA, USA).The most deranged laboratory values within 72 h of this specimen collection were recorded, including complete blood count (measured with an automated hematology analyzer, MC560, Pushikang Biological Technology Co., Ltd., Hangzhou, China), liver and renal function panels, lipid profile, myocardial injury markers, NT-proBNP, procalcitonin (measured with a fluorescence immunoassay system, Triage^®^ MeterPro, Alere Inc., San Diego, CA, USA), and blood lactate (GEM Premier 4000, Instrumentation Laboratory, Bedford, MA, USA). Pathogen identification results, nadir MAP, and the presence of tachyarrhythmia were also documented. (5) Interventions and complications: Invasive procedures and complications occurring from surgery until IFDs diagnosis. (6) Medications: Dosage and duration of vasoactive agents and administration of broad-spectrum antibiotics. (7) Outcomes: Length of ICU stay, total hospital stay, and 28-day mortality.

### 2.5. Statistical Analysis

Data analysis was performed using SPSS (version 27.0) and R software (version 4.4.0). Variables with >30% missing data were excluded; otherwise, missing values were imputed using multiple imputation with the mice R package (version 3.15.0). Normally distributed continuous variables were expressed as mean ± standard deviation (SD), while non-normally distributed variables were expressed as median and interquartile range (IQR). Categorical variables were presented as frequencies and percentages (%).

All candidate variables were entered into a LASSO regression model for feature selection using the glmnet package. Ten-fold cross-validation was used to determine the optimal regularization parameter (lambda.min). Variables selected by LASSO were then included in a multivariate logistic regression analysis to identify independent risk factors for short-term mortality. A nomogram was constructed to visualize the predictive model.

The model’s discriminative ability was evaluated using the ROC curve and the calculated AUC. Internal validation was performed using 1000 bootstrap resamples. Model calibration was assessed with a calibration curve and the Hosmer-Lemeshow goodness-of-fit test. The clinical utility of the model was evaluated using DCA and CIC. A two-sided *p*-value below 0.05 was considered statistically significant.

### 2.6. Ethical Statement

This retrospective study was approved by the Ethics Committee of QLH. The requirement for informed consent was waived due to the retrospective nature and use of de-identified data.

## 3. Results

### 3.1. Baseline Characteristics

Between January 2020 and December 2023, 8569 patients were admitted to the cardiac surgery ICU postoperatively. Of these, 98 adult patients met the inclusion criteria for IFDs. The overall incidence of postoperative IFDs was 1.14% (98/8569). The 28-day mortality rate among patients with IFDs was 42.9% (42/98), accounting for 0.49% (42/8569) of the total postoperative mortality in this cohort. The sites of infection included the lungs (*n* = 57), bloodstream (*n* = 28), both lungs and bloodstream (*n* = 12), and both lungs and urinary tract (*n* = 1).

The baseline, intraoperative, and postoperative characteristics of the study cohort are summarized in Table 1a–c. Among preoperative variables, a history of smoking was significantly more prevalent in non-survivors than in survivors (*p* < 0.001). No significant differences were observed between the two groups in terms of other baseline characteristics, including age, sex, BMI, or comorbidities such as diabetes, hypertension, and pulmonary hypertension. Left ventricular ejection fraction was also comparable. Intraoperative factors, including the type of surgical procedure, operative time, cardiopulmonary bypass duration, and transfusion rates, did not differ significantly between the groups. Postoperatively, non-survivors exhibited significantly higher SOFA and APACHE II, indicating a greater severity of illness (*p* < 0.001). This group also had a higher incidence of adverse outcomes, such as hypotension (MAP below 70 mmHg), tachyarrhythmia, and acute kidney injury, with a correspondingly greater need for renal replacement therapy. Prolonged mechanical ventilation (>48 h) was also more common among non-survivors.

### 3.2. Results of LASSO-Logistic Regression Analysis

**Figure 2:**
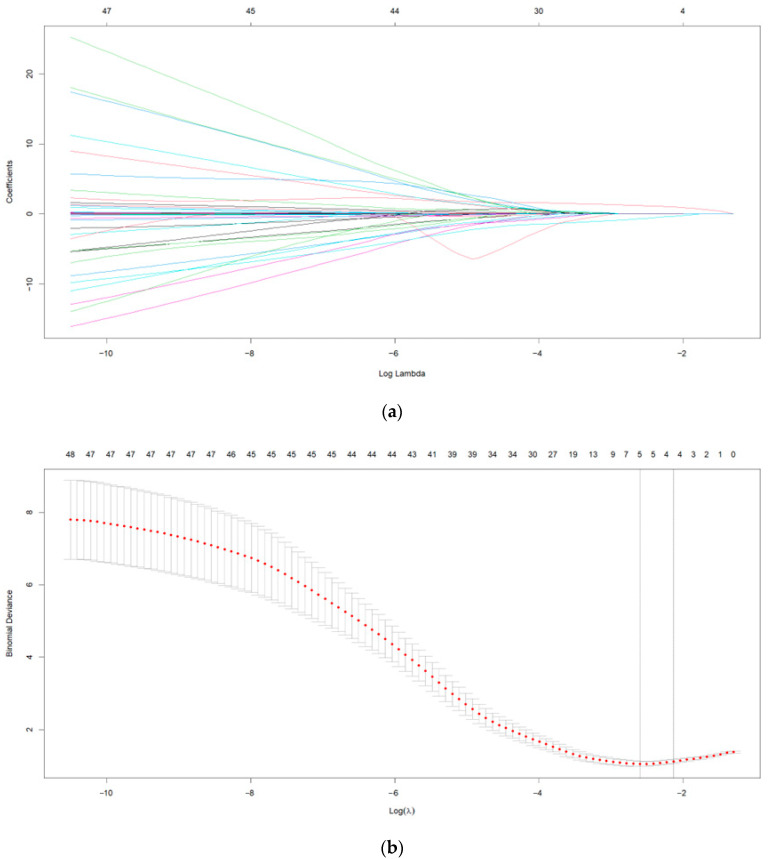
(**a**) LASSO Regression Coefficient Trajectories. (**b**) Ten-Fold Cross-Validation Curve. In the figure, the two dashed lines from left to right represent lambda.min and lambda.1se, respectively. Lambda.min corresponds to the λ value at the minimum mean squared error, while lambda.1se represents the λ value at one standard error from the minimum mean squared error.

Dimensionality reduction of processed risk factors was performed using LASSO regression to extract the most important predictors and avoid overfitting. The optimal parameter (lambda) in the LASSO model was selected using cross-validation based on the minimum standard. The minimum lambda value (lambda_min) was chosen as the optimal value for the model (Figure 2a,b). LASSO logistic regression analysis identified history of smoking, MAP below 70 mmHg, and tachyarrhythmia as significant risk factors (*p* < 0.05), with odds ratios (OR) of 6.11, 5.57, and 4.04, respectively, indicating that these factors significantly increased the risk of adverse outcomes. In preliminary multivariate logistic regression analysis, the *p*-value for the Apache II score was 0.627, which was much higher than 0.05, indicating that it was not an independent risk factor after controlling for other variables. Moreover, its 95% confidence interval crossed 1 (OR = 1.03, 95% CI: 0.93–1.13), further suggesting weak predictive power. To improve the statistical stability of the model and avoid adding noise from non-significant variables, the Apache II score was excluded from the final model. After removing the Apache II score, the *p*-value for the SOFA score decreased from 0.247 to 0.046, reaching statistical significance (*p* < 0.05) and demonstrating that SOFA score became an independent risk factor in the updated model. In addition, the optimized model’s pseudo R^2^ increased to 0.3973, indicating improved explanatory power, with all other variables remaining significant, suggesting that model performance was not compromised. Therefore, the final model retained the SOFA score and excluded the Apache II score (Table 2).

### 3.3. Development and Validation of the Predictive Model

**Figure 3:**
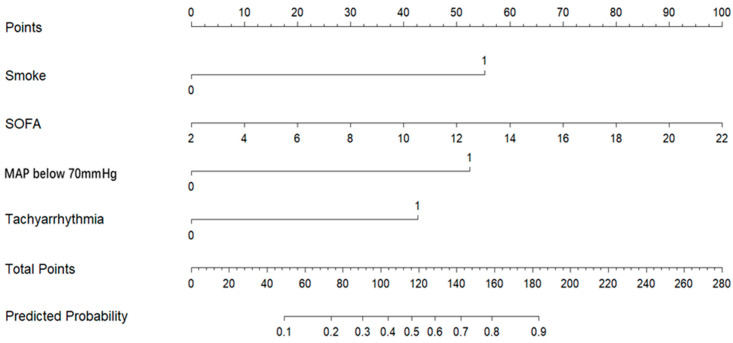
Nomogram Model Based on LASSO-Logistic Regression.

A nomogram was constructed based on the final logistic regression model to provide a visual tool for predicting the probability of short-term mortality (Figure 3).

**Figure 4:**
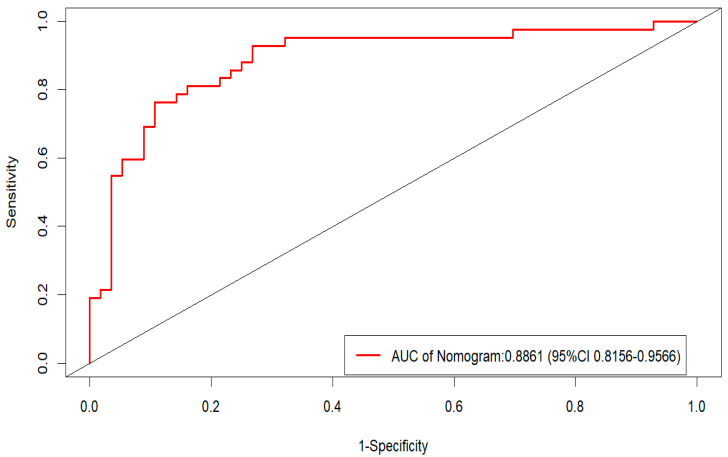
ROC Curve of the Predictive Model.

The model demonstrated excellent discrimination, with an AUC of 0.886 (95% CI: 0.816–0.957) (Figure 4). The optimal cutoff value was 0.420, yielding a sensitivity of 85.7% and a specificity of 80.4%. The Hosmer-Lemeshow goodness-of-fit test indicated good model calibration (χ^2^ = 7.97, *p* = 0.473).

**Figure 5:**
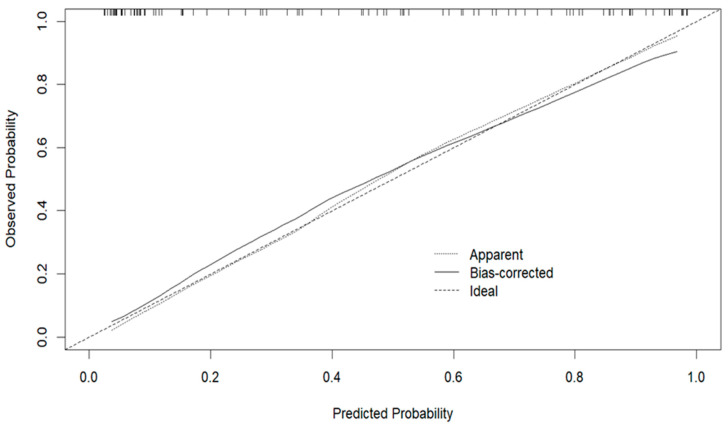
Calibration Curve of the Predictive Model.

Internal validation using 1000 bootstrap resamples confirmed the model’s robustness. The calibration curve showed strong agreement between predicted probabilities and actual mortality, with a mean absolute error of 0.023 (Figure 5).

**Figure 6:**
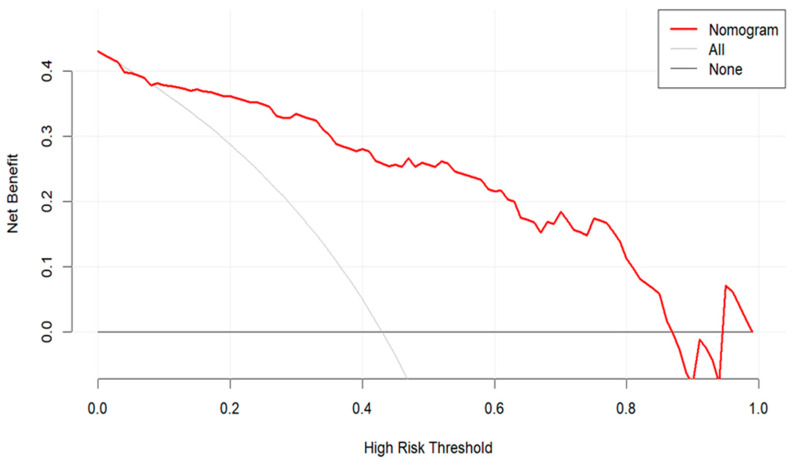
Decision Curve Analysis of the Predictive Model.

DCA demonstrated that the nomogram provided a net benefit over a wide range of threshold probabilities (0.10 to 0.87), indicating its clinical utility (Figure 6).

**Figure 7:**
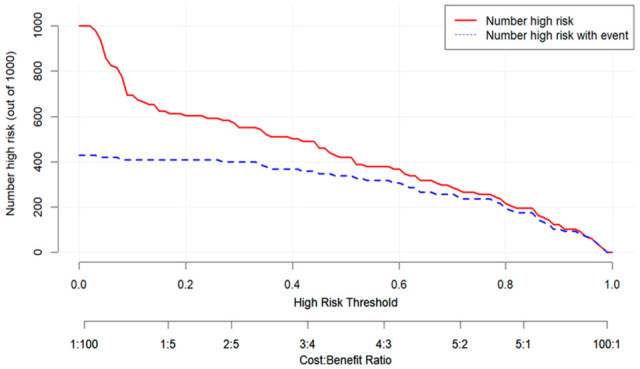
Clinical Impact Curve of the Predictive Model.

The clinical impact curve further illustrated a high concordance between the predicted number of high-risk patients and the number of observed deaths across different risk thresholds (Figure 7).

## 4. Discussion

Continuous advancements in cardiac surgery have significantly improved overall patient prognosis [8]. However, a subset of critically ill patients remains at high risk for adverse outcomes due to complications such as infection [9]. Postoperative infections in this setting are often linked to factors like cardiopulmonary bypass, therapeutic hypothermia, and the presence of prosthetic materials [10], making them one of the most frequent non-cardiac complications [1]. Critically ill patients typically exhibit compromised immune function and often require interventions such as mechanical ventilation, parenteral nutrition, and broad-spectrum antibiotics, all of which are established risk factors for opportunistic fungal infections [6].

In our cohort, the frequent use of broad-spectrum or combination antibiotic therapy (78.6%), prolonged mechanical ventilation, and parenteral nutrition underscore the high-risk profile of these patients. While these factors did not emerge as independent predictors in our multivariate analysis, their established association with IFDs warrants close clinical vigilance. These observations reinforce that IFDs is a major contributor to poor prognosis, prolonged hospitalization, and increased mortality risk in post-cardiac surgery patients.

Our analysis of 8569 patients over four years revealed an IFDs incidence of 1.14% and an associated mortality rate of 42.9%. These figures are consistent with previous reports [11,12] and align with findings from the multicenter AURORA study, which reported a 42.8% crude mortality for ICU-acquired IFDs [13]. This highlights that IFDs is a prevalent and lethal complication. Our study further identified four independent predictors of mortality in this specific population: a history of smoking, an elevated SOFA score, MAP below 70 mmHg, and tachyarrhythmia.

Smoking is a well-established independent risk factor for postoperative infections and mortality. A systematic review by Pourbaix et al. [14] confirmed that smoking significantly increases the risk of IFDs (RR = 1.41), suggesting a consistent effect across diverse patient populations. Our finding reinforces its role as a mortality predictor in patients who have already developed IFDs. The underlying pathophysiology may involve smoking-induced chronic lung disease, enhanced airway pathogen colonization, and impaired mucus clearance, predisposing patients to severe secondary infections.

The SOFA score is a cornerstone for assessing organ dysfunction in critical care. A nationwide study in Japan by Nishimoto et al. [15] found that the day-1 SOFA score effectively predicted mortality (AUROC = 0.75) in a mixed cardiac critical care cohort. Our study, focusing specifically on the post-cardiac surgery IFDs population, confirms that a higher SOFA score is a potent independent risk factor for death. This underscores the value of dynamic SOFA monitoring to identify high-risk individuals early. The exacerbated organ injury in these patients may stem from the “two-hit” insult of surgical trauma (e.g., ischemia-reperfusion from CPB) and subsequent fungal infection. Conversely, a study by Schoe et al. [16] using a large registry found that while SOFA had acceptable discrimination, other scores like APACHE IV performed better for mortality prediction in cardiac surgery patients. Therefore, while SOFA is an excellent tool for dynamic monitoring in our specific cohort, a more comprehensive prognostic assessment might incorporate additional scoring systems.

Furthermore, we identified MAP below 70 mmHg and tachyarrhythmia as independent risk factors. Infection is a common precipitant for acute heart failure (AHF) [17]. A large sepsis study by Chen Q et al. [18] demonstrated a U-shaped relationship between MAP and mortality, with a risk inflection point around 70–82 mmHg. Our finding aligns with this, emphasizing the critical need to maintain adequate organ perfusion pressure to mitigate organ failure in the face of fungal infection. Most cardiac surgery patients have pre-existing cardiac dysfunction. We observed no significant differences in conventional immune markers between our groups, suggesting these markers may not fully capture the “functional immunosuppression” state in these patients [19]. Based on this, we propose a “dangerous amplifying effect” between fungal infection and heart failure: infection-driven inflammation exacerbates cardiac dysfunction, while heart failure-induced hypoperfusion and organ congestion impair the immune response, creating a vicious cycle. Tachyarrhythmias, particularly postoperative atrial fibrillation (POAF), fit into this cycle. A meta-analysis showed POAF significantly increases long-term mortality and stroke risk [20]. More specific to our context, recent evidence suggests POAF is also an independent risk factor for mortality in patients with fungal bloodstream infections [21], supporting our hypothesis of an “infection-arrhythmia-death” cascade.

## 5. Limitations and Perspectives

This study has several limitations. First, as a single-center retrospective study, its relatively small sample size may limit the model’s generalizability. Second, the lack of external validation means that multicenter studies are required to confirm our findings and the model’s stability. Third, potential variations in diagnostic and therapeutic strategies over the four-year study period may have introduced bias.

For future perspectives, expanding the dataset by collaborating with other regional cardiac centers and incorporating data from public databases (e.g., MIMIC-IV) could help refine and validate the model. Furthermore, future research should not only focus on prognosis but also aim to develop a predictive model for the incidence of IFDs itself. Such efforts could enable preventative strategies, ultimately reducing the burden of IFDs and improving patient outcomes. These studies will also provide a platform to further investigate our proposed “dangerous amplifying effect” between fungal infection and heart failure.

## 6. Conclusions

A history of smoking, an elevated SOFA score, MAP below 70 mmHg, and tachyarrhythmia are independent risk factors for short-term mortality in patients with IFDs following cardiac surgery. The nomogram developed in this study provides a practical and accurate tool for risk stratification and may help guide clinical decision-making in this high-risk population.

## Figures and Tables

**Table 1:** (a). Preoperative Characteristics. (b). Intraoperative Characteristics. (c). Postoperative Characteristics.

Variable	Overall	Survivors	Non-Survivors	*p*-Value
(a)				
	(*N* = 98)	(*N* = 56)	(*N* = 42)	
Age (years, mean ± SD)	63.1 ± 11.1	63.9 ± 9.4	62.1 ± 13.0	0.51
Male, *n* (%)	53 (54.1%)	29 (51.8%)	24 (57.1%)	0.62
BMI (kg/m^2^, mean ± SD)	24.26 ± 4.31	24.40 ± 4.18	24.06 ± 4.53	0.7
Smoking, *n* (%)	48 (49.0%)	14 (25.0%)	34 (81.0%)	<0.001
Diabetes mellitus, *n* (%)	25 (25.5%)	13 (23.2%)	12 (28.6%)	0.56
Hypertension, *n* (%)	62 (63.3%)	33 (58.9%)	29 (69.0%)	0.32
LVEF [IQR]	0.58 (0.45,0.63)	0.59 (0.48,0.63)	0.55 (0.41,0.62)	0.65
NYHA class III–IV, *n* (%)	93 (94.90%)	54 (96.43%)	39 (92.86%)	0.65
Pulmonary hypertension, median [IQR]	29.5 [24.0–45.8]	28.5 [22.8–42.0]	30.0 [27.0–46.8]	0.42
(b)				
Type of surgery, *n* (%)				0.95
– Isolated CABG	30 (30.6%)	16 (28.6%)	14 (33.3%)	
– Valve surgery (repair or replacement)	20 (20.4%)	11 (19.6%)	9 (21.4%)	
– Combined Valve + CABG surgery	16 (16.3%)	9 (16.1%)	7 (16.7%)	
– Thoracic aortic surgery	24 (24.5%)	16 (28.6%)	8 (19.0%)	
– Congenital heart surgery	2 (2.0%)	1 (1.8%)	1 (2.4%)	
– Others	6 (6.1%)	3 (5.4%)	3 (7.1%)	
Operation time (min, Mean ± SD)	400.05 ± 133.19	401.43 ± 122.45	398.21 ± 147.80	0.91
Cardiopulmonary bypass time (min, mean ± SD)	202.00 (163.50,261.50)	199.50 (164.75,268.50)	202.00 (154.00,233.50)	0.57
Blood transfusion (yes, *n*%)	94 (95.92%)	52 (92.86%)	42 (100.00%)	0.13
(c)				
SOFA score, median [IQR]	11.0 [8.0–15.0]	9.5 [7.0–12.0]	14.5 [11.0–17.0]	<0.001
APACHE II, median [IQR]	24.0 [18.0–29.0]	20.0 [16.0–24.8]	28.5 [25.0–38.0]	<0.001
Use of ECMO or IABP, *n* (%)	18(18.4%)	10(17.9%)	8(19.0%)	0.88
MAP below 70 mmHg, *n* (%)	29 (29.6%)	7 (12.5%)	22 (52.4%)	<0.001
Tachyarrhythmia, *n* (%)	31 (31.6%)	11 (19.6%)	20 (47.6%)	0.004
AKI, *n* (%)	69 (70.4%)	32 (57.1%)	37 (88.1%)	0.002
RRT required, *n* (%)	38 (38.8%)	15 (26.8%)	23 (54.8%)	0.007
Hepatic dysfunction, *n* (%)	69 (70.4%)	35 (62.5%)	34 (81.0%)	0.06
Invasive mechanical ventilation > 48 h, *n* (%)	84 (85.7%)	43 (76.8%)	41 (97.6%)	0.006
Combination antibiotic therapy (≥2 agents), *n* (%)	76 (77.6%)	41 (73.2%)	35 (83.3%)	0.24
ICU stay > 7 days, *n* (%)	87 (88.8%)	47 (83.9%)	40 (95.2%)	0.08

**Table 2:** Multivariate Logistic Regression Analysis of Short-term Mortality in Cardiac Surgery Patients with Invasive Fungal Diseases.

Variable	β	SE	Wald Z	*p*-Value	Odds Ratio (95% CI)
Intercept	−4.1299	1.0222	−4.04	<0.0001	-
Smoke	1.8100	0.5863	3.09	0.0020	6.11 (1.94–19.28)
SOFA	0.1636	0.0819	2.00	0.0457	1.18 (1.00–1.38)
MAP below 70 mmHg	1.7174	0.6373	2.69	0.0070	5.57 (1.60–19.42)
Tachyarrhythmia	1.3960	0.6095	2.29	0.0220	4.04 (1.22–13.34)

## Data Availability

The original contributions presented in this study are included in the article. Further inquiries can be directed to the corresponding author.
